# Hyaluronic Acid Induction Promotes the Differentiation of Human Neural Crest-like Cells into Periodontal Ligament Stem-like Cells

**DOI:** 10.3390/cells12232743

**Published:** 2023-11-30

**Authors:** M. Anas Alhasan, Atsushi Tomokiyo, Sayuri Hamano, Hideki Sugii, Taiga Ono, Keita Ipposhi, Kozue Yamashita, Bara Mardini, Fumiko Minowa, Hidefumi Maeda

**Affiliations:** 1 Department of Endodontology and Operative Dentistry, Faculty of Dental Science, Kyushu University, Fukuoka 812-8582, Japan; anas@dent.kyushu-u.ac.jp (M.A.A.); shamano@dent.kyushu-u.ac.jp (S.H.); sugii@dent.kyushu-u.ac.jp (H.S.); tono@dent.kyushu-u.ac.jp (T.O.); ipposhi@dent.kyushu-u.ac.jp (K.I.); kozue3947@gmail.com (K.Y.); bara.mardini@dent.kyushu-u.ac.jp (B.M.); fminowa@dent.kyushu-u.ac.jp (F.M.); hide@dent.kyushu-u.ac.jp (H.M.); 2 Department of Restorative Dentistry, Faculty of Dental Medicine, Hokkaido University, Kita13 Nishi7, Kita-ku, Sapporo 060-8586, Japan; 3 OBT Research Center, Faculty of Dental Science, Kyushu University, Fukuoka 812-8582, Japan; 4 Department of Endodontology, Kyushu University Hospital, Fukuoka 812-8582, Japan

**Keywords:** periodontal ligament, stem cells, neural crest cells, hyaluronic acid-CD44 signalling, electrospun nanofibrous membranes

## Abstract

Periodontal ligament (PDL) stem-like cells (PDLSCs) are promising for regeneration of the periodontium because they demonstrate multipotency, high proliferative capacity, and the potential to regenerate bone, cementum, and PDL tissue. However, the transplantation of autologous PDLSCs is restricted by limited availability. Since PDLSCs are derived from neural crest cells (NCs) and NCs persist in adult PDL tissue, we devised to promote the regeneration of the periodontium by activating NCs to differentiate into PDLSCs. SK-N-SH cells, a neuroblastoma cell line that reportedly has NC-like features, seeded on the extracellular matrix of PDL cells for 2 weeks, resulted in the significant upregulation of PDL marker expression. SK-N-SH cell-derived PDLSCs (SK-PDLSCs) presented phenotypic characteristics comparable to induced pluripotent stem cell (iPSC)-derived PDLSCs (iPDLSCs). The expression levels of various hyaluronic acid (HA)-related genes were upregulated in iPDLSCs and SK-PDLSCs compared with iPSC-derived NCs and SK-N-SH cells, respectively. The knockdown of *CD44* in SK-N-SH cells significantly inhibited their ability to differentiate into SK-PDLSCs, while low-molecular HA (LMWHA) induction enhanced SK-PDLSC differentiation. Our findings suggest that SK-N-SH cells could be applied as a new model to induce the differentiation of NCs into PDLSCs and that the LMWHA–CD44 relationship is important for the differentiation of NCs into PDLSCs.

## 1. Introduction

Periodontal tissue comprises the gingiva, cementum, alveolar bone, and the periodontal ligament (PDL). The PDL is the fibrous connective tissue that covers the tooth root and connects it to the alveolar bone [1]. The successive management of periodontal lesions involves cooperative treatment of the PDL, cementum, and alveolar bone [2]. However, tooth loss can occur when the PDL is damaged beyond repair, which is frequent in deep caries, trauma, and periodontitis. As periodontitis progresses, the resultant inflammation damages the structure of the PDL, making it difficult to repair lost PDL tissue [3] and eventually leading to tooth loss.

Stem cells are important for tissue repair and regeneration. The periodontal tissue contains PDL stem cells (PDLSCs) that demonstrate multipotency, high proliferative capacity, and the potential to regenerate bone, cementum, and PDL tissue [4,5]. PDLSCs are considered more suitable than other dental stem cells for periodontal tissue regeneration [6]. Practically, the transplantation of autologous human PDLSCs has reduced the defect area and increased bone density [7]. However, the population of PDLSCs in PDL tissue is thought to be very low [8], and since the isolation of autologous PDLSCs requires tooth extraction, it is difficult to obtain sufficient numbers of cells for clinical applications.

Neural crest (NC) cells are a population of migratory and multipotent stem cells that are initially generated at the junction of the epidermal and neural ectoderm [9]. NC cells migrate to their destination in the embryo and differentiate into various cell and tissue types, including sensory, autonomic, and enteric ganglia within the peripheral nervous system, the adrenal medulla, melanocytes, and a range of skeletal, connective, adipose, and endocrine cell types [10]. While the periodontal tissue originates from the cranial NC, some NC cells remain there and give rise to PDLSCs [11,12]. NC cells residing in somatic tissues have the potential to differentiate into various other stem cell types, making them promising candidates for tissue regeneration [13]. Theoretically, periodontal tissue regeneration could be promoted by inducing NC cells within periodontal tissue to differentiate into PDLSCs. However, the challenge in acquiring large numbers of NC cells is a principal factor limiting their application in regenerative studies.

In 2006, Shinya Yamanaka’s group developed a method to reprogram somatic cells to acquire properties similar to those of embryonic stem cells (ESCs). Those cells, named induced pluripotent stem cells (iPSCs), overcome many of the limitations of ESCs and can be differentiated into many cell types, revolutionising the field of regenerative medicine [14]. Several previous studies reported the generation of iPSC-derived NC-like cells (iNCs) from iPSCs [15,16,17]. Additionally, we recently developed a protocol to induce human iNCs to differentiate into iNC-derived PDLSC-like cells (iPDLSCs), which exhibit the high expression of PDL-related markers, high proliferative ability, mesenchymal stem cell (MSC)-related marker expression, and mesenchymal-lineage differentiation potential [18]. Therefore, experiments with iPDLSCs would be expected to clarify which factors are needed to induce iNCs to differentiate into iPDLSCs. However, this exact differentiation model is not ideal, given the long culture required to first differentiate iPSCs into iNCs.

Neuroblastoma, which develops from the central nervous system and is derived from NC cells, is the most common extracranial solid tumour in infants. The SK-N-SH neuroblastoma cell line was established from a human stage IV neuroblastoma in 1970 [19]. SK-N-SH is distinguished by the presence of three morphologically and biochemically distinct cell phenotypes: neuroblastic (N-type), non-neuronal substrate-adherent (S-type), and an intermediate phenotype between N and S (I-type) [20]. Based on these characteristics, SK-N-SH cells are used in various research areas, including drug resistance, tumour development, gene transfection, enzyme activity, phospholipid homeostasis, and cellular differentiation [21,22].

The aim of this study was to investigate whether SK-N-SH cells could serve as an alternative differentiation model for iNCs, to identify factors that regulate the differentiation and promote the transformation of NC-like cells into PDLSC-like cells, and to develop a new scaffold that includes these factors. Here, we report that SK-N-SH cells co-cultured with human PDL cells-derived extracellular matrix (ECM) presented PDLSC-like characteristics. This differentiation process was regulated by the relationship between low-molecular-weight hyaluronic acid (LMWHA) and CD44. Additionally, LMWHA-containing electrospun nanofibrous membranes also promoted their PDLSC differentiation.

## 2. Materials and Methods

### 2.1. Cell Culture

Human PDLCs (HPDLCs) were isolated from a 17-year-old female who visited Kyushu University Hospital for tooth extraction, as described previously [23] Briefly, PDL tissues were stripped from the root surface of the extracted teeth and incubated in the presence of collagenase and trypsin (FUJIFILM Wako, Osaka, Japan) for 20 min at 37 °C. Suspended cells were harvested and centrifuged at 1000 rpm for 5 min, followed by seeding on culture dishes in alpha-minimum essential medium (α-MEM; Gibco-BRL, Grand Island, NY, USA) containing 10% foetal bovine serum (FBS; Sigma-Aldrich, St. Louis, MO, USA), 50 U/mL penicillin, and 50 mg/mL streptomycin (FUJIFILM Wako, Osaka, Japan) (control medium, CM) at 37 °C in a humidified atmosphere of 5% CO_2_ and 95% air. Cells from passages 4 through 8 were used in the experiments. Human SK-N-SH cells were obtained from RIKEN (RCB No. 0426; Saitama, Japan) and were cultured in CM and maintained in the same 37 °C incubator. All procedures were performed in compliance with the Research Ethics Committee, Faculty of Dentistry, Kyushu University (approval number: 2-115).

### 2.2. PDLSC Induction

The PDLSC-induction protocol used in this study was based on a method previously described by Hamano et al. [18]. Briefly, HPDLCs were cultured to confluence in 24-well plates, followed by treatment with 2% EDTA (Nacalai Tesque, Kyoto, Japan) to detach cells and obtain plates coated with HPDLC-derived ECM (HPDLCs-ECM). SK-N-SH cells were seeded at a density of 2 × 10^4^ cells/well into HPDLCs-ECM-coated 24-well plates in α-MEM containing 10% FBS. After 2 weeks of culture, the adherent cells were designated as SK-N-SH cell-derived PDLSC-like cells (SK-PDLSCs).

### 2.3. Osteoblastic Differentiation

SK-N-SH cells and SK-PDLSCs were seeded at 2 × 10^4^ cells/well into 24-well plates and cultured in α-MEM (Gibco-BRL, Grand Island, NY, USA) containing 10% FBS (Sigma-Aldrich, St. Louis, MO, USA). After the cells reached confluence, the culture medium was changed to osteoblastic differentiation medium (OM): α-MEM containing 10% FBS and supplemented with 2 mM β-glycerophosphate (Sigma-Aldrich, St. Louis, MO, USA), and 50 mg/mL ascorbic acid (Nacalai Tesque, Kyoto, Japan). After 3 weeks, the cells were collected for the isolation of total RNA for reverse transcriptase (RT)-PCR or were fixed in 10% formalin (FUJIFILM Wako, Osaka, Japan), washed with distilled water, and stained with 0.3% Alizarin Red S for calcium detection. Stained cells were imaged using a Keyence BZ-9000 microscope (Keyence, Osaka, Japan). Measurements and the quantification of Alizarin Red S-positive areas were performed using BZ-X Analyzer Software (V: 1.1.2.4, Keyence, Osaka, Japan).

### 2.4. Adipocytic Differentiation

SK-N-SH cells and SK-PDLSCs were cultured at 2 × 10^4^ cells/well into 24-well plates in α-MEM (Gibco-BRL, Grand Island, NY, USA) containing 10% FBS (Sigma-Aldrich, St. Louis, MO, USA). After the cells reached confluence, the culture medium was changed to adipocytic differentiation medium (AM): α-MEM containing 10% FBS supplemented with 0.1 mM ascorbic acid (FUJIFILM Wako, Osaka, Japan), 0.5 mM methylisobutylmethylxanthine (Sigma-Aldrich, St. Louis, MO, USA), 0.5 mM hydrocortisone (Sigma-Aldrich, St. Louis, MO, USA, and 60 mM indomethacin (Sigma-Aldrich, St. Louis, MO, USA). After 3 weeks, the cells were collected for the isolation of total RNA for RT-PCR or were fixed with 10% formalin (FUJIFILM Wako, Osaka, Japan), washed with distilled water, and stained with 0.3% oil red O for lipid droplet detection. Stained cells were imaged using a Keyence BZ-9000 microscope (Keyence, Osaka, Japan).

### 2.5. Quantitative RT-PCR (qRT-PCR)

The expression levels of commonly probed PDL-related genes (*COL1A1*, *FBN1*, *OPG*, *POSTIN*, *αSMA*, *PLAP1*, and *TNMD*), osteogenic genes (*BMP2*, *BSP*, *OPN*, and *RUNX2*), adipogenic genes (*ADIPSIN*, *ADIPOQ*, *FSP27*, and *LEP*), and HA-related genes (*CD44*, *ACAN*, *ITIH3*, *HAS3*, and *HAPLN1*) were analysed via qPCR. Briefly, cells were harvested for the isolation of total RNA using TRIzol reagent (Invitrogen, Invitrogen, Carlsbad, CA, USA), cDNA was synthesised using an ExScript RT reagent kit (Takara Bio, Shiga, Japan), and qPCR was performed using a KAPA Express Extract kit (Takara Bio, Shiga, Japan), each in accordance with the respective manufacturer’s instructions. The annealing temperature for all primers was 60 °C. Expression levels of target genes were calculated from ΔΔCt values. *β-actin* expression was used as an internal control, and qPCR results were normalised to the reference sample. Primer sequences were designed using the GenBank database, and a BLAST search of GenBank was performed to ensure primer sequence specificity. Specific primer sequences, annealing temperatures, and product sizes for PCR amplification of each gene are listed in Table 1.

### 2.6. Gene Expression Microarray

Total RNA was extracted from iNCs and iPDLSCs [18] using TRIzol (Invitrogen, Invitrogen, Carlsbad, CA, USA) reagent. RNA samples were quantified using an ND-1000 spectrophotometer (NanoDrop, Wilmington, DE, USA) and assessed for quality with an Agilent 2200 TapeStation (Agilent Technologies, Santa Clara, CA, USA). RNA was labelled using a one-colour Low Input QuickAmp Labeling Kit (Agilent Technologies) and hybridised to a SurePrint G3 Human Gene Expression Microarray v3 (8 × 60 Kb; Agilent Technologies). Relative hybridisation intensities and background hybridisation values were calculated using Agilent Feature Extraction Software version 10.7.1.1 (Agilent Technologies). After normalisation, the intensity data were filtered to exclude genes showing a <2-fold expression change between iNCs and iPDLSCs. Gene Ontology (GO) enrichment analysis was also performed using the Database for Annotation, Visualization and Integrated Discovery (DAVID) bioinformatics resources (http://david.ncifcrf.gov/, accessed on 10 April 2021). Enriched GO functions were identified on the basis of *p* values < 0.05.

### 2.7. Magnetic-Activated Cell Sorting (MACS) and Flow Cytometric Analysis

SK-N-SH cells were sorted via MACS using a bead-conjugated antibody against CD44 (Miltenyi Biotec, Bergisch Gladbach, Germany) in accordance with the manufacturer’s instructions. Briefly, 6 × 10^6^ SK-N-SH cells were suspended in Flow Cytometry Staining Buffer (R&D Systems, Minneapolis, MN, USA) and centrifuged at 300× *g* for 10 min, resuspended in 80 µL of MACS buffer (Miltenyi Biotec, Bergisch Gladbach, Germany) with 20 µL CD44 microbeads, and incubated in the dark at 4 °C for 15 min. Cells were then washed with MACS buffer, resuspended in 500 µL MACS buffer, and loaded into an LD column (Miltenyi Biotec, Bergisch Gladbach, Germany) placed in the magnetic field of a MidiMACS separator (Miltenyi Biotec, Bergisch Gladbach, Germany). Unlabelled cells (SK-CD44−) that passed through the column were collected in a tube. Labelled cells (SK-CD44+) that remained inside the column were flushed with buffer and collected in a different tube. Sorted cells were further cultured for 2 weeks on dishes coated with or without HPDLCs-ECM and then used for the qPCR measurement of PDL-related gene expression. For flow cytometric analysis, SK-N-SH cells were detached from plates, quantified, transferred to tubes (4 × 10^5^ cells/tube), incubated with phycoerythrin (PE)-conjugated anti-mouse/human CD44 antibodies (Miltenyi Biotec, Bergisch Gladbach, Germany) for 1 h at 4 °C, and washed with flow cytometry buffer (R&D Systems, Minneapolis, MN, USA). The percentage of positive cells was measured using an EC800 Cell Analyzer (Sony, Tokyo, Japan). Data were further analysed using Eclipse software (V:1.3.6, Sony, Tokyo, Japan).

### 2.8. Small Interfering RNA (siRNA) Transfection

SK-N-SH cells were reverse transfected with custom human *CD44* siRNA (siCD44 Stable, Sense Strand (5′ to 3′) GAAUAUAACCUGCCGCUUUUU, Antisense Strand (5′ to 3′) AAAGCGGCAGGUUAUAUUCUU, Dharmacon, Lafayette, CO, USA) or human control siRNA (siSTABLE Non-Targeting sRNA #1, D-001700-01-05, Dharmacon, Lafayette, CO, USA) using Lipofectamine RNAiMAX (Invitrogen). Briefly, an siRNA–lipid complex comprising 10 pmol siRNA and 3 µL Lipofectamine RNAiMAX in 50 µL Opti-MEM (Invitrogen) was prepared and added to each well of a 24-well plate. SK-N-SH cells (2 × 10^4^ cells/well) were diluted, suspended in 450 µL Opti-MEM I containing 10% FBS, and added to the wells containing the siRNA–lipid complex. After 24 h, the medium was changed to CM, the plates were incubated for 2 weeks, and cells were collected to isolate total RNA for qPCR.

### 2.9. HA Stimulation

High-molecular-weight HA (HMWHA; FCH-200; Kikkoman, Tokyo, Japan) and low-molecular-weight HA (LMWHA; FCH-80, Kikkoman, Tokyo, Japan) were used in this study. SK-N-SH cells were seeded in wells in CM containing 0, 0.1, 0.3, 0.6, or 1.0 mg/mL HMWHA or LMWHA or in wells coated with or without HPDLCs-ECM in CM or CM containing 0.3 mg/mL HMWHA or LMWHA. Every 2 days, the culture medium was replaced with fresh medium of the same composition. Alternatively, SK-N-SH cells were seeded in wells coated with or without HPDLCs-ECM in CM with or without 2.0 mg/mL LMWHA for 2 days, after which the medium was changed to CM and was replaced with fresh CM every 2 days thereafter. Following 2 weeks of culture, the expression of *CD44* and PDL-related genes was investigated using qPCR.

### 2.10. Fabrication of LMWHA-Containing Electrospun Nanofibrous Membranes

The electrospun nanofibre membranes were fabricated by Nanon-04 (MECC, Fukuoka, Japan). The polymer solution was prepared by dissolving 11 wt% of polycaprolactone (PCL; Mw = 80,000; Sigma-Aldrich, St. Louis, MO, USA) in 2,2,2-trifluoroethanol (TFE; Nacalai Tesque, Kyoto, Japan) at room temperature. Different amounts of LMWHA (0, 1.1, or 2.2%) were mixed with the solution. These mixtures were transferred to a 5 mL plastic syringe attached to a stainless-steel needle (TERUMO, Tokyo, Japan) with an inner diameter of 0.22 mm via a polytetrafluoroethylene Teflon tube. The rotating drum collector was wrapped with a cellulose-based composite and kept at a 150 mm distance from the stainless-steel needle. An electric potential of 20 kV was applied between the needle and the rotating drum. The dope solution was pumped out at a rate of 1.0 mL/h. The collection speed of the collector was 100 rpm. The relative humidity inside the electrospinning chamber was maintained using a dehumidifier (F-YC80ZLX; Panasonic, Osaka, Japan), and the temperature was kept from 24.1 to 26.4 °C. These electrospun membranes were air dried for 24 h to remove the residual solvent.

### 2.11. Scanning Electron Microscopy

In advance of scanning electron microscopy (SEM) analysis, the electrospun nanofibre membranes were coated with gold-palladium. Their surface was observed via SEM (S-3400N, Hitachi High-Technologies Co., Tokyo, Japan) at an acceleration voltage of 15 kV.

### 2.12. Enzyme-Linked Immunosorbent Assay (ELISA)

To quantify the concentration of HA released from LMWHA-containing electrospun nanofibrous membranes, a sensitive two-site ELISA was performed. The electrospun nanofibrous membranes containing 0, 1.1, or 2.2 wt% LMWHA were cut using sterilised scissors in the shape of a square that had the same surface area of 1.9 cm^2^ as the wells of a 24-well plate. These membranes were immersed in CM for 2 days, and the quantity of HA released from membranes was measured using a commercially available ELISA kit (Hyaluronan Quantification Kit, PG research, Tokyo, Japan) in accordance with the manufacturer’s instructions. Briefly, 100 µL of HA Coating Solution was added to the wells of the 96-well plate for 1 h. Wells were then washed gently with Wash Buffer four times, and a blocking buffer was added for 30 min. Following one wash, the samples, as well as the standards, were added to the wells with an equivalent amount of Biotin-HABP and incubated together for 1 h. Wells were then washed again, and HRP-Avidin was added to the wells for 1 h. After a final wash, a Substrate Solution was added to the wells, and they were left for an additional 30 min in the dark. A Stop Solution was added, and the absorbance was measured using a microplate reader at an absorbance of 450 nm. All samples and standards were measured in triplicate.

### 2.13. Culturing SK-N-SH Cells with LMWHA-Containing Electrospun Nanofibrous Membranes

SK-N-SH cells were seeded in wells coated with or without HPDLCs-ECM in CM. Then, the electrospun nanofibrous membranes containing 0, 1.1, or 2.2% LMWHA were cut by sterilised scissors in the shape of a square that had the same surface area of 1.9 cm^2^ as the wells of a 24-well plate, and were placed above the cells. The culture medium was replaced with fresh CM every 2 days. Following 2 weeks of culture, the expression of *CD44* and PDL-related genes was investigated using qPCR.

### 2.14. Statistical Analysis

All values were normalised to *β-actin* expression and are presented as means ± standard deviations. Group comparisons were performed using one-way analysis of variance, followed by Tukey’s multiple comparison post-hoc test. Student’s unpaired *t* tests were performed for comparisons of two mean values. A *p*-value < 0.05 was considered to indicate statistical significance.

## 3. Results

### 3.1. Differentiation of SK-N-SH Cells into PDLSCs

**Figure 1:**
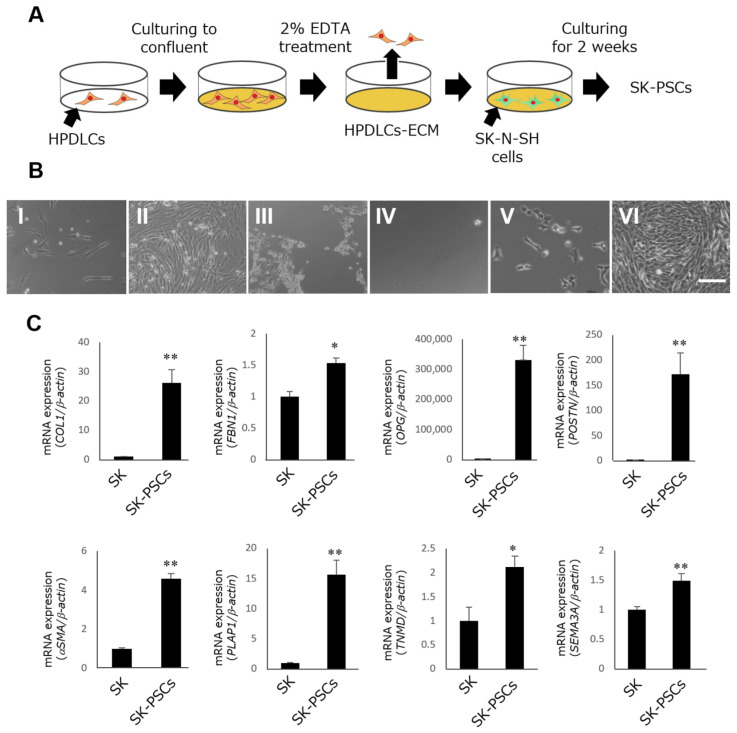
Differentiation of SK-N-SH cells into SK-PDLSCs. (**A**) Schematic diagram of the generation of SK-PDLSCs from SK-N-SH cells. (**B**) Representative phase-contrast microscopic images of HPDLCs and SK-N-SH cells. HPDLCs were cultured in CM for 1 day (**I**) and 4 days (**II**). HPDLCs were detached (**III**) and completely removed (**IV**) from wells via EDTA treatment, leaving wells coated with HPDLCs-ECM. SK-N-SH cells were cultured in HPDLCs-ECM-coated wells in CM for 3 days (**V**) and 2 weeks (**VI**). Scale bar = 100 µm. (**C**) qPCR analysis of expression levels of eight PDL-related genes (*COL1A1*, *FBN1*, *OPG*, *POSTN*, *αSMA*, *PLAP1*, *TNMD*, and *SEMA3A)* in SK-N-SH cells and SK-PDLSCs. *β-actin* was used as an internal standard. Data are expressed as the mean ± standard deviation (n = 3); * *p* < 0.05, ** *p* < 0.01.

HPDLCs exhibited the typical shapes of fibroblasts during culture in CM (Figure 1B(I)). They proliferated and fully covered the surface of the wells after 4 days of culture (Figure 1B(II)). Complete detachment of HPDLCs from the wells using EDTA treatment (Figure 1B(III)) was confirmed via phase-contrast microscopy (Figure 1B(IV)). After 3 days of culture on HPDLCs-ECM-coated wells, SK-N-SH cells exhibited spindle shapes (Figure 1B(V)). They also proliferated and completely covered the surface of the wells after 2 weeks of culture (Figure 1B(VI)). SK-PDLSCs were found to have significantly higher expression of PDL-related genes (*COL1A1*, *FBN1*, *OPG*, *POSTIN*, *αSMA*, *PLAP1*, *TNMD*, and *SEMA3*) compared with SK-N-SH cells (Figure 1C).

**Figure 2:**
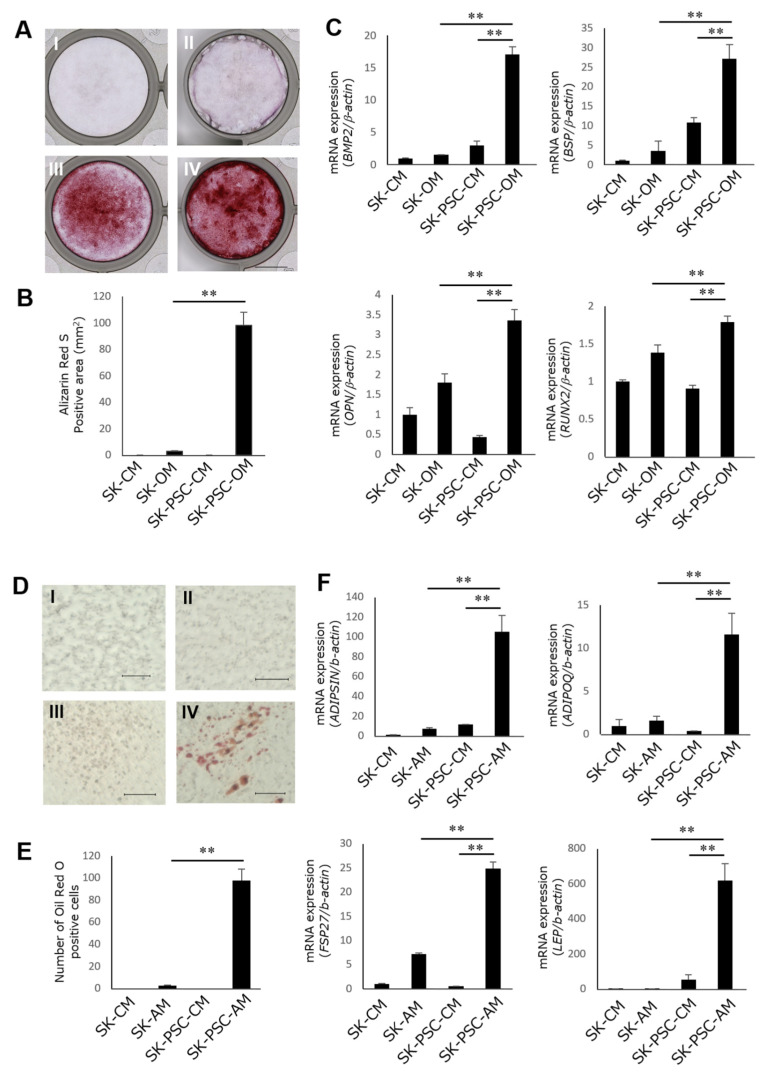
Osteoblastic and adipocytic differentiation of SK-PDLSCs. (**A**) Representative images of Alizarin Red S-stained SK-N-SH cells (**I**,**III**) and SK-PDLSCs (**II**,**IV**) after 3 weeks of culture in CM (**I**,**II**) or OM (**III**,**IV**). Scale bar = 0.5 cm. (**B**) The area of Alizarin Red S-positive staining in SK-N-SH and SK-PDLSCs cultured in CM or OM for 3 weeks. (**C**) qPCR analysis of expression levels of four osteoblast-associated genes (*BMP2*, *BSP*, *OPN*, and *RUNX2*) in SK-N-SH cells and SK-PDLSCs after 3 weeks of culture in CM or OM. (**D**) Representative images of oil red O-stained SK-N-SH cells (**I**,**III**) and SK-PDLSCs (**II**,**IV**) after 3 weeks of culture in CM (**I**,**II**) or AM (**III**,**IV**). Scale bars = 100 µm. (**E**) Numbers of oil red O-positive cells in SK-N-SH and SK-PDLSCs cultured in CM or AM for 3 weeks. (**F**) qPCR analysis of expression levels of four adipocyte-associated genes (*ADIPSIN*, *ADIPOQ*, *FSP27*, and *LEP*) in SK-N-SH cells and SK-PDLSCs after 3 weeks of culture in CM or AM. *β-Actin* was used as an internal standard. Data are expressed as the mean ± standard deviation (n = 3); ** *p* < 0.01. SK-CM, SK-N-SH cells cultured in CM; SK-OM, SK-N-SH cells cultured in OM; SK-PSC-CM, SK-PDLSCs cultured in CM; SK-PSC-OM, SK-PDLSCs cultured in OM; SK-AM, SK-N-SH cells cultured in AM; SK-PSC-AM, SK-PDLSCs cultured in AM.

Next, we tested the multipotency of the SK-PDLSCs by investigating their ability to differentiate into osteoblasts and adipocytes. SK-N-SH cells and SK-PDLSCs exposed to CM showed no positive staining for Alizarin Red S (Figure 2A(I,II)). While SK-N-SH cells cultured in osteoblastic differentiation medium (OM) showed some Alizarin Red S-positive staining (Figure 2A(III)), it was not as strong as that of SK-PDLSCs cultured in OM (Figure 2A(IV)). There was statistically significant difference in Alizarin Red S-positive areas between cultures (Figure 2B). The qPCR results also showed that the expression levels of osteoblast-related genes (*BMB2*, *BSP*, *OPN*, and *RUNX2*) were significantly higher in SK-PDLSCs cultured in OM than in SK-N-SH cells cultured in CM or OM or in SK-PDLSCs cultured in CM (Figure 2C). While SK-N-SH cells cultured in CM or adipocytic differentiation medium (AM), as well as SK-PDLSCs cultured in CM, did not produce oil red O-positive fatty lipids (Figure 2C(I–III)), SK-PDLSCs cultured in AM produced clear fat lipids (Figure 2D(IV)). There were statistically significant differences in the numbers of cells exhibiting lipid droplets between SK-PDLSCs in AM and SK-N-SH in AM (Figure 2E). The expression levels of adipocyte-related genes (*ADIPSIN*, *ADIPOQ*, *FSP27*, and *LEP*) were also significantly higher in SK-PDLSCs cultured in AM than in SK-N-SH cells cultured in CM or AM or in SK-PDLSCs cultured in CM (Figure 2F).

### 3.2. Genes Upregulated by Differentiation of NC Cells into PDLSCs

**Figure 3:**
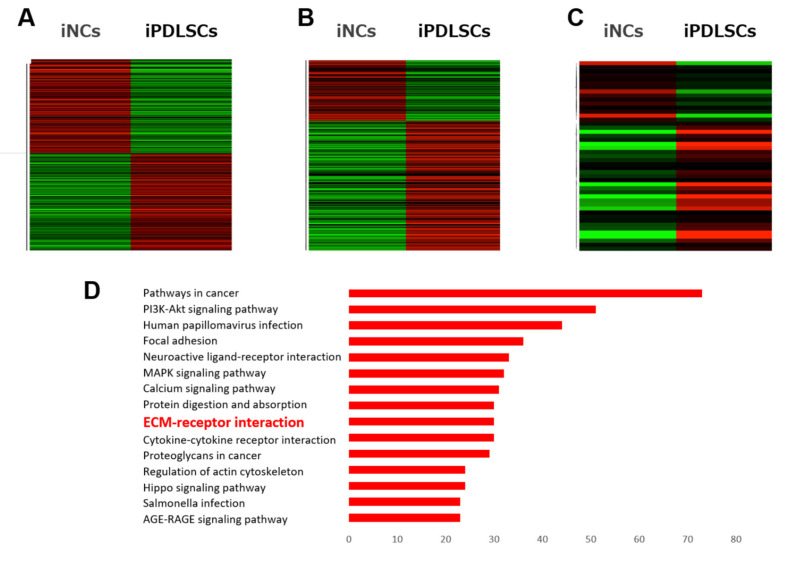

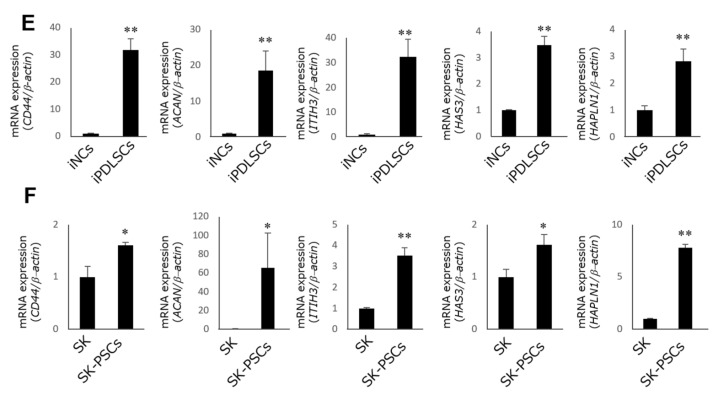
Upregulated expression of HA-related genes during the differentiation of iNCs into iPDLSCs. (**A**–**C**) Heat maps of gene expression data from the microarray analyses of iNCs and iPDLSCs for all genes (**A**), ECM-related genes (**B**), and HA-related genes (**C**). Upregulated genes are represented in red, and downregulated genes are represented in green. (**D**) GO enrichment analysis using the DAVID bioinformatics resources and based on >2-fold altered expression between iPDLSCs and iNCs. Data portray the top 15 significant terms ranked by the enrichment factor. The term ‘ECM-receptor interaction’ included 30 genes. (**E**,**F**) qPCR analysis of expression levels of five HA-related genes (*CD44*, *ACAN*, *ITIH3*, *HAS3*, and *HAPLN1*) in iNCs and iPDLSCs (**E**) and in SK-N-SH cells and SK-PDLSCs (F). *β-Actin* was used as an internal standard. Data are expressed as the mean ± standard deviation (n = 3); * *p* < 0.05, ** *p* < 0.01.

We compared gene expression in iNCs and iPDLSCs using the microarray analysis described in our previous report [18]. The ratio of genes that were upregulated and downregulated during the differentiation from iNC to iPDLSC was approximately 1:1 (Figure 3A). Interestingly, in iPDLSCs, more ECM-related genes (Figure 3B) were upregulated than downregulated, especially among the HA-related genes (Figure 3C). DAVID analysis revealed that the expression of 30 genes in the ECM–receptor interaction pathway was altered by >2-fold during the differentiation from iNCs to iPDLSCs, representing the ninth highest number of gene alterations of all pathways (Figure 3D). Based on these findings, we focused on the role of HA signalling in the differentiation process from iNCs to iPDLSCs. The expression levels of HA-related genes in iNCs and iPDLSCs were analysed using qPCR. Similar to the microarray results, HA-related genes were more highly expressed in iPDLSCs than in iNCs (Figure 3E). The expression of HA-related genes in SK-N-SH cells and SK-PDLSCs showed the same pattern: HA-related genes were more highly expressed in SK-PDLSCs than in SK-N-SH cells (Figure 3F).

### 3.3. Differentiation of CD44+ SK-N-SH Cells into PDLSCs

**Figure 4:**
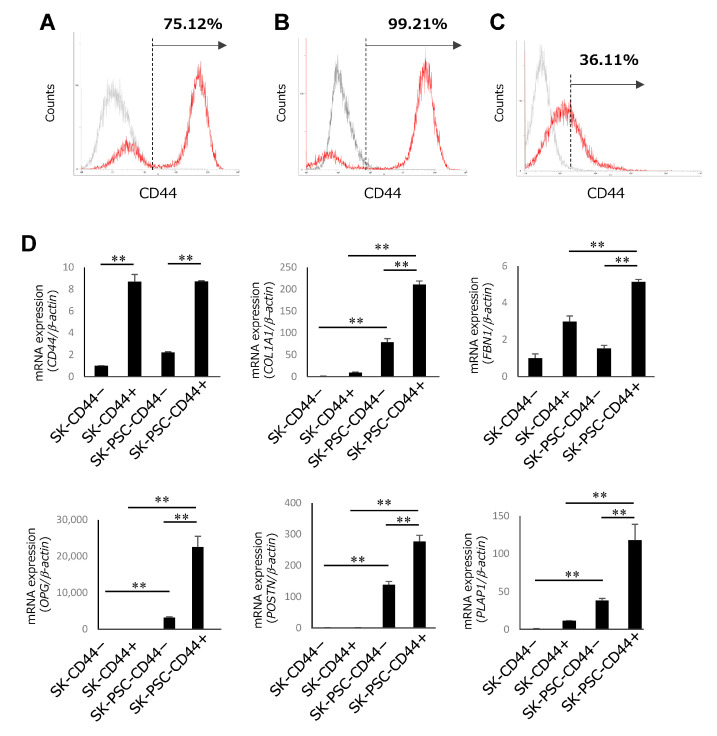
Effects of CD44 enrichment on the differentiation of SK-N-SH cells into SK-PDLSCs. (**A**–**C**) Flow cytometric analysis of CD44 expression in SK-N-SH cells (**A**), SK-CD44+ (**B**), and SK-CD44− (**C**) after 3 days of culture. Histograms show fluorescence-labelled cells expressing CD44 (red lines) and the isotype control (grey lines). (**D**) qPCR analysis of expression levels of *CD44* and five PDL-related genes (*COL1A1*, *FBN1*, *OPG*, *POSTN*, and *PLAP1*) in SK-CD44−, SK-CD44+, SK-PSC-CD44−, and SK-PSC-CD44+ after 2 weeks of culture. *β-Actin* was used as an internal standard. Data are expressed as the mean ± standard deviation (n = 3); ** *p* < 0.01.

CD44 is the major surface receptor for HA, and its activation induces the initiation of intracellular signalling pathways [24]. Therefore, purified CD44-expressing SK-N-SH cells were used to investigate the involvement of HA signalling in their differentiation into SK-PDLSCs. Flow cytometric analysis demonstrated that 75.12% of all SK-N-SH cells were positive for CD44 (Figure 4A). MACS purification increased the proportions of CD44+ SK-N-SH cells (SK-CD44+) to 99.21% (Figure 4B) and CD44-SK-N-SH cells (SK-CD44−) to 36.11% (Figure 4C). SK-CD44+ and SK-CD44− were further cultured on wells coated with or without HPDLCs-ECM. After 2 weeks of culture, these cells were named SK-PSC-CD44+ and SK-PSC-CD44−, respectively. SK-CD44+ and SK-PSC-CD44+ showed significantly higher expression of CD44 than SK-CD44− and SK-PSC-CD44− (Figure 4D). SK-PSC-CD44− and SK-PSC-CD44+ also showed upregulated expression of *COL1A1*, *OPG*, *POSTN*, and *PLAP1* compared with SK-CD44− and SK-CD44+, respectively. Expression levels of FBN1 were elevated in SK-PSC-CD44+ compared with that of SK- CD44+, but were not different between SK-CD44− and SK-PSC-CD44−. Expression levels of these five PDL-related genes were also increased in SK-PSC-CD44+ compared with those in SK-PSC-CD44−.

### 3.4. Differentiation of CD44-Suppressed SK-N-SH Cells into PDLSCs

**Figure 5:**
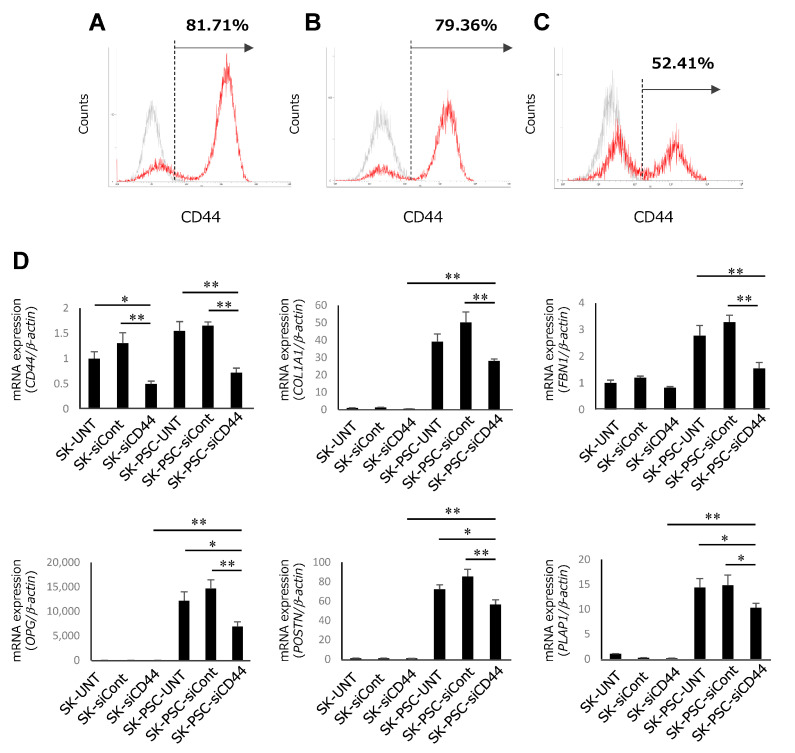
Effects of CD44 knockdown on the differentiation of SK-N-SH cells into SK-PDLSCs. (**A**–**C**) Flow cytometric analysis of CD44 expression in SK-UNT (**A**), SK-siCont (**B**), and SK-siCD44 (**C**) after 3 days of culture. Histograms show fluorescence-labelled cells expressing CD44 (red lines) and the isotype control (grey lines). (**D**) qPCR analysis of expression levels of *CD44* and five PDL-related genes (*COL1A1*, *FBN1*, *OPG*, *POSTN*, and *PLAP1*) in SK-UNT, SK-siCont, SK-siCD44, SK-PSC-UNT, SK-PSC-siCont, and SK-PSC-siCD44 after 2 weeks of culture. β-Actin was used as an internal standard. Data are expressed as the mean ± standard deviation (n = 3); * *p* < 0.05, ** *p* < 0.01.

To further investigate the function of HA signalling in the differentiation of SK-N-SH cells into SK-PDLSCs, CD44 knockdown was performed. SK-N-SH cells without siRNA transfection (SK-UNT) or with control siRNA transfection (SK-siCont) showed similar levels of CD44 expression (SK-UNT: 81.71%; SK-siCont: 79.36%; Figure 5A,B). By contrast, SK-N-SH cells transfected with CD44 siRNA (SK-siCD44) exhibited decreased expression of CD44 (52.41%) compared with SK-UNT and SK-siCont (Figure 5C). SK-UNT, SK-siCont, and SK-siCD44 were further cultured on wells coated with HPDLCs-ECM, becoming SK-PSC-UNT, SK-PSC-siCont, and SK-PSC-siCD44. After 2 weeks of culture, SK-siCD44 and SK-PSC-siCD44 exhibited significantly lower expression of CD44 compared with SK-UNT, SK-siCont, SK-PSC-UNT, and SK-PSC-siCont (Figure 5D). The levels of PDL-related gene expression in SK-PSC-siCD44 were higher than those in SK-siCD44 but were significantly lower than those in SK-PSC-UNT and SK-PSC-siCont.

### 3.5. Effect of Continuous HA Stimulation on Differentiation of SK-N-SH Cells into SK-PDLSCs

**Figure 6:**
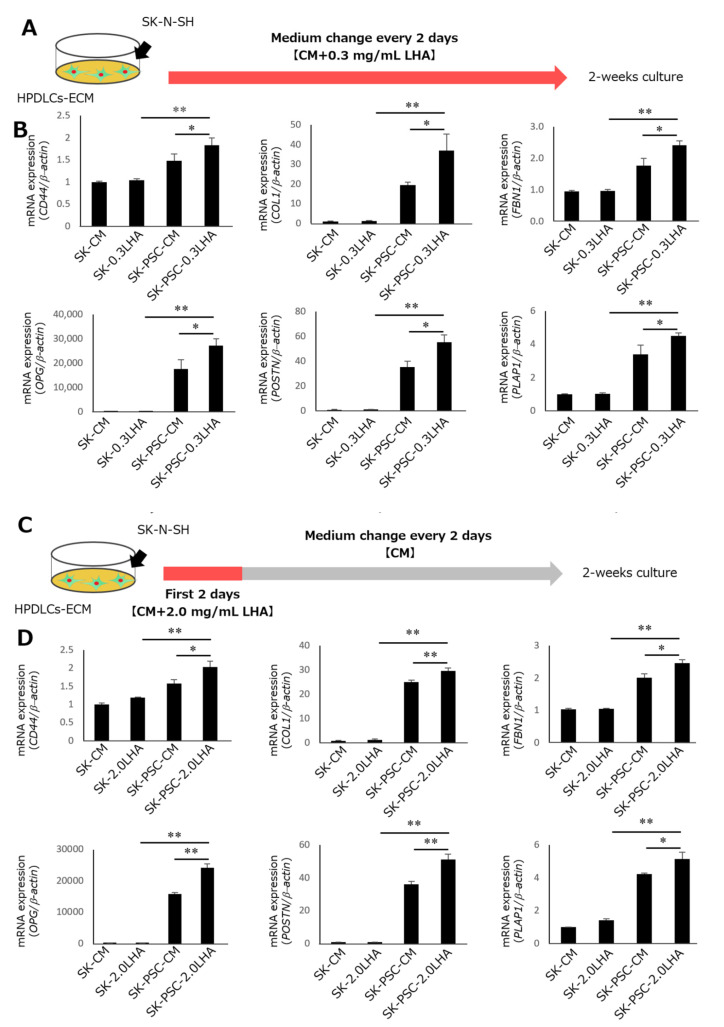
Effects of LMWHA stimulation on the differentiation of SK-N-SH cells into SK-PDLSCs. (**A**) Schematic diagram of continuous stimulation with LMWHA in SK-N-SH cells cultured on HPDLSCs-ECM. (**B**) qPCR analysis of expression levels of *CD44* and five PDL-related genes (*COL1A1*, *FBN1*, *OPG*, *POSTN*, and *PLAP1*) in SK+CM, SK+0.3LMWHA, SK-PSC-CM, and SK-PSC+0.3LMWHA after 2 weeks of culture. (**C**) Schematic diagram of initial stimulation with LMWHA in SK-N-SH cells cultured on HPDLSCs-ECM. (**D**) qPCR analysis of expression levels of *CD44* and five PDL-related genes (*COL1A1*, *FBN1*, *OPG*, *POSTN*, and *PLAP1*) in SK+CM, SK+2.0LMWHA, SK-PSC-CM, and SK-PSC+2.0LMWHA after 2 weeks of culture. β-Actin was used as an internal standard. Data expressed as the mean ± standard deviation (n = 3); * *p* < 0.05, ** *p* < 0.01. SK+CM, SK-N-SH cells without HA stimulation; SK+0.3LMWHA, SK-N-SH cells with continuous 0.3 mg/mL LMWHA stimulation; SK-PSC-CM, SK-N-SH cells cultured on HPDLSCs-ECM without LMWHA stimulation; SK-PSC+0.3LMWHA, SK-N-SH cells cultured on HPDLSCs-ECM with continuous 0.3 mg/mL LMWHA stimulation; SK+2.0LMWHA, SK-N-SH cells with initial 2.0 mg/mL LMWHA stimulation; SK-PSC+2.0LMWHA, SK-N-SH cells cultured on HPDLSCs-ECM with initial 2.0 mg/mL LMWHA stimulation.

The biological effects of HA depend on its molecular weight, which ranges from <10 kDa to >6000 kDa [25]. Therefore, we investigated the effects of continuous stimulation with HA at two different molecular weights (HMWHA and LMWHA) on the differentiation of SK-N-SH cells (Supplementary Figure S1A). After 2 weeks of the induction of SK-N-SH cells, stimulation with 0.1, 0.3, 0.6, or 1.0 mg/mL HMWHA did not increase the expression of either *COL1A1* or *POSTN* in SK-N-SH cells compared with that in cells cultured in CM (Supplementary Figure S1B). By contrast, stimulation with 0.1, 0.3, 0.6, and 1.0 mg/mL LMWHA significantly upregulated *COL1A1* expression in SK-N-SH cells (Supplementary Figure S1C). Additionally, treatment with 0.3, 0.6, and 1.0 mg/mL LMWHA also increased *POSTN* expression, most strongly at 0.3 mg/mL. Therefore, we used 0.3 mg/mL LMWHA stimulation for further experiments (Figure 6A). After 2 weeks of SK-N-SH cell culture without HPDLCs-ECM, stimulation with LMWHA did not increase the expression levels of *CD44* and PDL-related genes above those in unstimulated cells (Figure 6B). However, SK-N-SH cells cultured in the presence of HPDLCs-ECM and LMWHA showed significantly higher expression of these genes than the cells cultured in HPDLCs-ECM alone.

### 3.6. Effect of Initial LMWHA Stimulation on Differentiation of SK-N-SH Cells into SK-PDLSCs

To provide continuous stimulation with LMWHA, CM containing 0.3 mg/mL LMWHA was replaced every 2 days for 2 weeks. To test the effect of HA on the early stages of the differentiation, an initial LMWHA stimulation was performed, and cCM with only CM containing either 1.0 mg/mL or 2.0 mg/mL was added to the cells for the first 2 days of culture (Figure 6C). After 2 weeks of culture without HPDLCs-ECM, the expression levels of *CD44* and PDL-related genes were similar in unstimulated and initially LMWHA-stimulated SK-N-SH cells (Figure 6D). By contrast, in SK-PDLSCs cultured in the presence of HPDLCs-ECM, the initial stimulation with LMWHA led to the significantly higher expression of these genes.

### 3.7. Effect of LMWHA-Containing Electrospun Nanofibrous Membranes on Differentiation of SK-N-SH Cells into SK-PDLSCs

**Figure 7:**
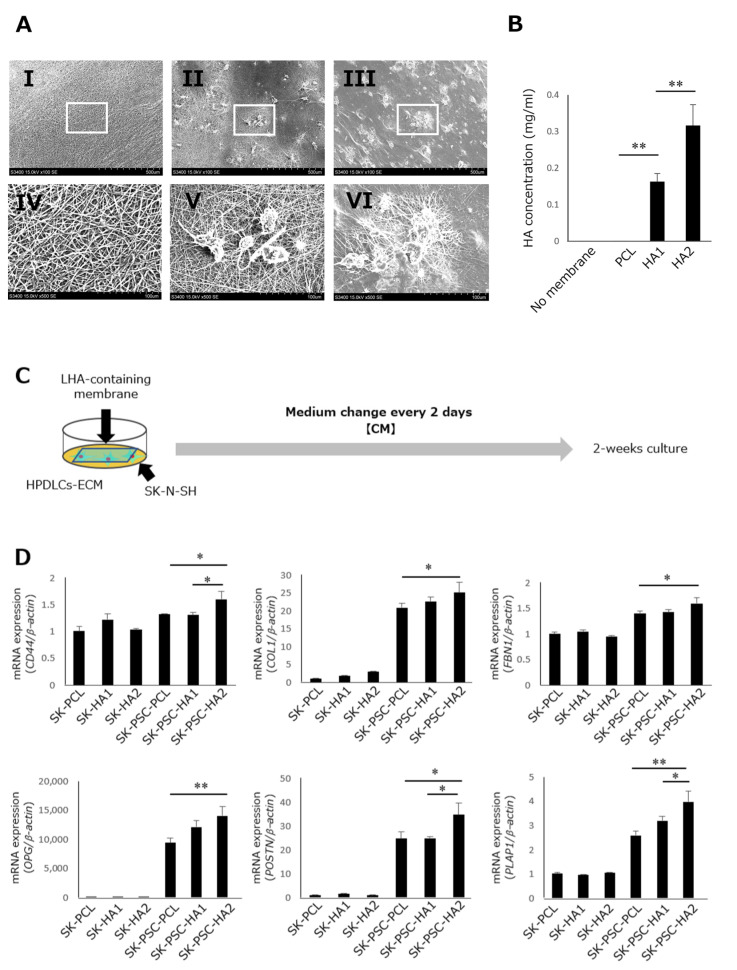
Effects of LMWHA-containing electrospun nanofibrous membranes on the differentiation of SK-N-SH cells into SK-PDLSCs. (**A**) SEM micrographs obtained from PCL (**I**,**IV**), H1 (**II**,**V**), and H2 (**III**,**VI**) membranes. Original magnifications: 100× (**I**–**III**) and 500× (**IV**–**VI**). White boxes in (**I**–**III**) indicate magnified areas. (**B**) HA concentration in CM incubated with PCL, HA1, and HA2 membrane. (**C**) Schematic diagram of the coculture of LMWHA-containing electrospun nanofibrous membranes with SK-N-SH cells. (**D**) qPCR analysis of expression levels of *CD44* and five PDL-related genes (*COL1A1*, *FBN1*, *OPG*, *POSTN*, and *PLAP1*) in SK+PCL, SK+HA1, SK+HA2, SK-PSC+PCL, SK-PSC+HA1, and SK-PSC+HA2 after 2 weeks of culture. *β-Actin* was used as an internal standard. Data are expressed as the mean ± standard deviation (n = 3); * *p* < 0.05, ** *p* < 0.01. PCL, polycaprolactone; H1, PCL with 1.1% LMWHA; H2, PCL with 2.2% LMWHA; SK+PCL, SK-N-SH cells with PCL membrane; SK+H1, SK-N-SH cells with H1 membrane, SK+H2, SK-N-SH cells with H2 membrane; SK-PSC+PCL, SK-N-SH cells cultured on HPDLSCs-ECM with PCL membrane; SK-PSC+H1, SK-N-SH cells cultured on HPDLSCs-ECM with H1 membrane; SK-PSC+H2, SK-N-SH cells cultured on HPDLSCs-ECM with H2 membrane.

SEM analysis showed that the fibres were uniformly thick without defects or damage in the electrospun nanofibrous membrane without LMWHA (Figure 7A(I,IV)). The electrospun nanofibrous membranes with 1.1 and 2.2% LMWHA also revealed the uniformity of thickness in their fibres; however, many polygonal particles were observed on their surface at 100× (Figure 7A(II,III)) and 500× magnifications (Figure 7A(V,VI)). An ELISA assay confirmed the release of HA from LMWHA-containing electrospun nanofibrous membranes with 1.1% LMWHA releasing 0.163 ± 0.032 mg/mL of HA and with 2.2% LMWHA releasing 0.316 ± 0.074 mg/mL of HA (Figure 7B). By contrast, a tiny amount of HA was detected in the CM (0.94 × 10^−4^ ± 0.37 × 10^−5^ mg/mL) and CM incubated with control PCL membranes (1.16 × 10^−4^ ± 0.16 ×10^−4^ mg/mL). Next, SK-N-SH cells were co-cultured with these membranes to investigate their potential to induce differentiation into SK-PDLSCs. At first, the wells were coated with HPDLC-ECM, and then, SK-N-SH cells were seeded on the ECM directly using the same method described on Figure 1. Therefore SK-N-SH cells can directly contact the ECM component to differentiate into SK-PDLSCs. To further improve the differentiation, the electrospun membranes containing HA were placed above the cells floating in the medium, while the. HA component in the membrane dissolves gradually to stimulate the HA–CD44 relationship and further improve the differentiation. After 2 weeks of culture without HPDLCs-ECM, SK-N-SH cells exposed to membranes with 1.1 and 2.2% LMWHA showed almost the same pattern in the expression of *CD44* and PDL-related genes as the cells with the control membrane (Figure 7D). The cells cultured in the presence of HPDLCs-ECM and 1.1% LMWHA membrane showed a slightly higher expression of these genes than the cells cultured in HPDLCs-ECM and the control membrane. However, the 2.2% LMWHA membrane led to the significantly higher expression of these genes in SK-PDLSCs cultured in the presence of HPDLCs-ECM.

## 4. Discussion

In this study, we established a new model to produce PDLSC-like cells from SK-N-SH cells. Previously, we successfully differentiated human iPSCs into iNCs, which were further differentiated into iPDLSCs [18] However, differentiating iPSCs into iPDLSCs requires many processes, long incubation, and differentiation periods; therefore, the establishment of a new model that avoids the use of iPSCs and iNCs could shorten the production of PDLSCs from NC cells. SK-N-SH cells comprise three types of cells: N-type, S-type, and I-type. S-type cells have been suggested to possess the features of embryonic NC precursor cells because they differentiate towards NC lineages, such as glial, meningeal, and melanocytic cells [26]. Boeva et al. demonstrated that a subclone of SK-N-SH cells, SH-EP cells, exhibited an S-type phenotype, with features closely resembling the super-enhancer landscapes of two primary human NC cell types [27]. Additionally, Ross et al. suggested that I-type cells were also NC cells because of their potential to generate progeny that showed neuronal characteristics and to replicate multipotent I-type progeny [28]. These results suggested that the SK-N-SH cell line includes a subpopulation of cells with an NC-like phenotype.

We found that SK-PDLSCs, like iPDLSCs, showed higher expression of PDL-related marker genes than SK-N-SH cells. Our previous study also showed that PDL-related marker genes were not upregulated in non-NC cells, even those cultured with HPDLCs-ECM [18], suggesting that SK-N-SH cells could be used as a substitute for iNCs in experiments to induce the differentiation of NC cells into PDLSCs. Additionally, SK-PDLSCs showed higher potential to differentiate into osteoblasts and adipocytes than SK-N-SH cells. These findings were similar to our previous study, which found that iPDLSCs formed more mineralised nodules and lipid droplets than iNCs after osteoblastic and adipocytic inductions [18]. PDLSCs are a type of MSC derived from cranial NC cells. Von Levetzow et al. suggested that NC cells give rise to not only mesenchymal cells, but also other types of cells, including neurons, glial cells, melanocytes, and endocrine cells; however, they tend to differentiate into mesenchymal-lineage cells after their commitment toward MSCs [29]. Therefore, we expected that iNCs and SK-N-SH cells cultured on HPDLCs-ECM would become committed toward PDLSCs and show higher osteoblastic and adipocytic differentiation potential compared with cells cultured without HPDLCs-ECM.

Next, we aimed to identify the factors that regulate the differentiation of NCs into PDLSCs to activate the remaining NCs and involve them in the regeneration of the periodontium. Microarray analysis of iNCs was essential to uncover genes significantly upregulated during differentiation that might control the differentiation process; therefore, HA-related genes were found to be upregulated in iPDLSCs compared to iNCs. It is worth mentioning that the comparison of all gene expression for SK-PDLSCs should also be important to investigate their characteristics. However, since these cells were derived from different patients, their expression patterns may be fundamentally different. Ideally, iPS cells would better be established from the same person from whom SK-N-SH cells were isolated to compare all gene expression data for iPDLSCs derived from them to SK-PDLSCs through microarray analysis, but this would be extremely difficult. Since HA-related genes in both iNCs and SK-N-SH cells were similar as proven based on qPCR results, we continued to the next steps since our goal to identify genes controlling the differentiation process was fulfilled. Data from microarray and qPCR analyses demonstrated that various HA-related genes were upregulated as iNCs and SK-N-SH cells differentiated into iPDLSCs and SK-PDLSCs, respectively. HA is a biodegradable, hydrophilic, and highly biocompatible glycosaminoglycan that is found throughout the human body. HA plays crucial roles in regulating the differentiation, proliferation, migration, adhesion, angiogenesis, and inflammation responses in various cell types [30]. In the periodontium, HA is synthesised by fibroblasts, keratinocytes, cementoblasts, and osteoblasts; however, its expression is stronger in non-mineralised tissues, including the gingiva and PDL, than in mineralised tissues, such as the cementum and alveolar bone [31]. Hence, we focused on the function of HA as a possible key factor in the differentiation process of NCs into PDLSCs. CD44 is a transmembrane receptor for HA, and the direct binding of the two is known to initiate various intracellular signalling cascades [30]. The CD44 antigen is expressed in periodontal tissues and the HA–CD44 interaction has been associated with periodontal ligament (PDL) cell proliferation and mineralisation activities [32]. In a previous report, HA–CD44 interactions were reported to mediate contractility and migration in periodontal ligament cells in which HA induction resulted in increased contractility and the reduced migration speed of human PDL cells. However, when CD44 knockout mouse cells were used, the contractility of the cells was unaffected by the induction with HA while their migration speed was enhanced [33]. However, the role of HA signalling in the differentiation of NCs into PDLSCs is not known yet. Therefore, we purified CD44+ and CD44- subpopulations of SK-N-SH cells to investigate the involvement of the pathway regulated by CD44 in the differentiation into SK-PDLSCs. Ours results demonstrated that SK-CD44+ cultured on HPDLCs-ECM showed a higher potential to differentiate into SK-PDLSCs compared with SK-CD44− This finding was consistent with previous reports. For example, the CD44+ fractions of enteroendocrine and tuft cells were found to be capable of generating intestinal organoids, whereas CD44− cells completely failed to generate them [34]. Moreover, myoblast progenitors from CD44−/− mice exhibited the attenuated potential to differentiate into myoblasts and form myotubes [35]. In this study, the involvement of CD44 in SK-N-SH cells was confirmed by inhibiting CD44 via siRNA-mediated knockdown, which decreased the potential of SK-N-SH cells to differentiate into SK-PDLSCs compared with that of untransfected and control siRNA-transfected cells. Based on these findings, we determined that CD44 is a key regulator for the differentiation of SK-N-SH cells into SK-PDLSCs.

Previous studies reported that HA molecules of different molecular weights have distinct effects on the differentiation of various types of stem/progenitor cells. Low-molecular-weight (LMW) HA promoted the proliferation of rabbit bone marrow-derived MSCs, while high-molecular-weight (HMW) HA induced their osteoblastic differentiation [36]. The osteoblastic differentiation of human bone marrow-derived MSCs was promoted by culturing them with a LMWHA-based hydrogel [37]. LMWHA also promoted the differentiation of human umbilical cord-derived MSC pneumocytes [38]. These results suggest that different stem cell types express HA with specific molecular weights required to induce their differentiation. Hence, we investigated the effects of HA at two different sizes (HMWHA and LMWHA) on the differentiation of SK-N-SH into SK-PDLSCs. The effects of HA on differentiation are also reportedly concentration-dependent [39]; hence, we tested HMWHA and LMWHA at concentrations of 0.1, 0.3, 0.6, and 1.0 mg/mL. COLIA1 is the main type of collagen in PDL tissue, and PDLSCs possess higher collagen-forming capacity than MSCs [40]. POSTN is the major ECM component of PDL tissue, playing important roles in the cross-linkage and distribution of collagen [41], and it may be useful as a periodontal regeneration marker [42]. Therefore, we utilised the high expression of *COLIA1* and *POSTN* as indicators of the effectiveness of HA treatment and identified 0.3 mg/mL LMWHA as the optimal concentration. Then, we examined the timing of stimulation with LMWHA to optimise the differentiation method. Intra-articular injection of HA is a widely used treatment for patients with knee osteoarthritis [43]. With regard to systemic HA treatment in patients, it is typically administered at a high concentration, and is gradually degraded and metabolised. To simulate in vivo conditions, we performed an initial stimulation with LMWHA and discovered that treatment with 2.0 mg/mL LMWHA for 2 days only induced the PDLSC-like differentiation of SK-N-SH cells. This result was consistent with a previous report that mouse bone marrow-derived MSCs were successfully differentiated into adipocytes following an initial 3 days of culture in induction medium [44].

A drug delivery system (DDS) is defined as a formulation or device that allows for the introduction of a therapeutic agent into the body and improves its efficacy and safety by controlling the release of the drug and reducing systemic side effects. A variety of DDSs have been produced over the past few decades, including prodrugs, permeation enhancers, gels, ointments, and liposome nanocarriers [45]. Among these, the use of synthetic biodegradable polyesters in DDSs is of great interest because they are hydrolysed into safe metabolic by-products in the body and do not require a second surgery for their removal after complete drug release. Electrospinning is a versatile technique by which polymer nanofibres can be produced using an electrostatically driven jet of polymer solution. Electrospun nanofibres have attracted attention in tissue engineering research because the fabricated nanofibre membrane can mimic the organised fibrous architecture observed in the ECM, and the high surface area of the membrane is favourable for cell attachment and DDSs [46]. Therefore, we applied electrospinning to produce the biodegradable scaffold-releasing LMWHA and induce the differentiation of NCs into PDLSCs. PCL, a semi-crystalline and biodegradable polymer, has been widely and successfully used in medical devices and tissue engineering because of its chemical and biological properties, physicochemical state, degradability, mechanical strength, and ease of electrospinning [47]. Jiang et al. transplanted composite PCL/polyethylene glycol nanofibrous membranes with bone-grafting materials, which led to the significant formation of tooth-supporting mineralised tissue with PDL-like tissue [48]. Additionally, Farag et al. produced electrospun PCL membranes with PDLSC-derived ECM and demonstrated their potential to support periodontal attachment [49]. Based on these results, PCL was used for electrospun scaffold fabrication in this study. Our results demonstrated that 1.1% and 2.2% LMWHA were successfully incorporated into the electrospun PCL membranes, which was consistent with a previous study reporting that electrospun PCL membranes could contain up to 5% HA [50]. SK-N-SH cells cultured in the presence of HPDLCs-ECM and the 2.2% LMWHA membrane exhibited significantly higher expression of CD44 and PDL-related genes than the cells cultured in HPDLCs-ECM and a control PCL membrane. The reason for using the concentration of membranes containing 2.2% LMWHA is that it released 0.316 ± 0.074 mg/mL HA, which was very similar to that which increased the expression of PDL-related genes in SK-N-SH cells under continuous stimulation. Meanwhile, concentrations lower than this (1.1%) have resulted in the reduced upregulation of PDL-related genes. Additionally, we tried to fabricate electrospun membranes with higher concentration of HA. However, concentrations higher than that were difficult to fabricate successfully because of the higher viscosity of the HA solution. However, the amount of LMWHA released from the membrane is expected to decrease over time, and our study demonstrated that an initial stimulation with 2.0 mg/mL LMWHA was sufficient to upregulate the expression of *CD44* and PDL-related genes. Therefore, further research is needed to produce an electrospun scaffold that can release approximately 2.0 mg/mL LMWHA immediately after implantation into the body. The morphology of the electrospun membrane may also have an effect on the differentiation of SK-N-SH cells, in addition to the membrane component. Therefore, our next plan is to study the effect of the morphology of the membrane, such as its thickness and porosity. Our ultimate goal is to develop a membrane that can efficiently induce the differentiation of NC cells into PDLSCs by applying its components and morphology.

While this study provides valuable insights into the role of the LMWHA–CD44 relationship in the differentiation of PDLSCs, it is important to acknowledge its limitations. One such limitation is the use of a single internal control gene (*β-actin*) for PCR. Recent guidelines suggest the use of at least two validated reference genes for normalisation. The use of a single reference gene, in this case, may lead to potential inaccuracies in the results due to the variable expression of the reference gene under different experimental conditions. Another limitation is that we used HPDLC-ECM from only one patient. PDL cells can produce various types of ECM, such as collagen, fibronectin, proteoglycan, osteopontin, bone sialoprotein, and osteonectin. Additionally, the composition of the ECM varies depending on factors, such as sex, age, and the type of tissue. We analysed the inductive effects of human periodontal ligament cells (HPDLCs) from various patients (detailed data not included). We discovered that the HPDLCs obtained from a 17-year-old patient were the most potent. While other HPDLCs also demonstrated inductive effects, they were less pronounced compared to the 17-year-old patient’s HPDLCs. Finally, our study does not account for the complex interactions in a living organism that could influence the efficacy of our treatment. We found that the CD44–HA interaction is essential for PDLSC differentiation, and thus, our next step is to stimulate this interaction using a scaffold system (LMWHA-containing electrospun nanofibrous membranes) to be applied alone (without SK-N-SH cells) in vivo to induce periodontal tissue regeneration by promoting the differentiation of the remaining NCs within periodontal tissue to differentiate into PDLSCs, which is needed to address this limitation.

## 5. Conclusions

In conclusion, our overall findings demonstrated that SK-N-SH cells have potential utility as a new model for the induced differentiation of NCs into PDLSCs. Additionally, we determined that the LMWHA–CD44 relationship is important for the differentiation of SK-N-SH cells into SK-PDLSCs and that the activation of HA signalling at the initial phase of differentiation is especially important for SK-PDLSCs. The electrospun PCL membrane with 2.2% LMWHA would have the potential to promote the differentiation of SK-N-SH cells into SK-PDLSCs. We anticipate that the application of an LMWHA-releasing biodegradable scaffold will regenerate destructed the periodontium by activating and differentiating NCs within the PDL and increasing the number of PDLSCs.

## Figures and Tables

**Table 1:** GenBank ID, primer sequences, and product sizes for quantitative reverse transcription polymerase chain reaction.

Target Gene (Abbreviation)	Gene Bank ID	Forward (Top) and Reverse (Bottom) Primer Sequences	Size of Amplified Products (bp)
Aggrecan (ACAN)	NM_001411096.1	AGTCCTCAAGCCTCCTGTACTCA	185
		CGGGAAGTGGCGGTAACA	
Adiponectin (ADIPOQ)	NM_001177800.2	CAGGAAACCACGACTCAAGGG	200
		CCGGTTTCACCGATGTCTCC	
Angiopoietin Like 4 (ANGPTL4)	NM_001039667.3	CCACTTGGGACCAGGATCAC	123
		GGATGGAGCGGAAGTACTGG	
ADIPSIN	NM_001317335.2	GTGCGCGGAGAGCAATCG	131
		AGATCCCGGGCTTCTTGCG	
Actin Alpha 2, Smooth Muscle (αSMA)	NM_001613.4	GACAATGGCTCTGGGCTCTGTA	147
		CTGTGCTTCGTCACCCACGTA	
B-actin	NM_001101.5	TGGCACCCAGCACAATGAA	89
		CTAAGTCATAGTCCGCCTAGAAGCA	
Bone Morphogenetic Protein 2 (BMP2)	NM_001200.4	TCCACTAATCATGCCATTGTTCA	73
		GGGACACAGCATGCCTTAGGA	
Bone Sialoprotein (BSP)	MN_004967.3	CTGGCACAGGGTATACAGGGTTAG	181
		ACTGGTGCCGTTTATGCCTTG	
CD44	NM_001202555.2	CTGCAGGTATGGGTTCATAG	123
		ATATGTGTCATACTGGGAGGTG	
Collagen Type I Alpha 1 (COL1A1)	NM_000088	CCCGGGTTTCAGAGACAACTTC	148
		TCCACATGCTTTATTCCAGCAATC	
Fibrillin-1 (FBN1)	NM_000138.4	AAGTGATCCACTGTGTGCCAACTC	189
		TGCACCTATGGAACCATGTGATAG	
Fat-Specific Protein FSP27 (FSP27)	NM_001199551.2	CTTCATTGGCTGCCTGAACG	195
		CCTTCCTCCGTAGCATCGAG	
Inter-Alpha-Trypsin Inhibitor Heavy Chain 3 (ITIH3)	NM_001392025.1	GCCTACCTCACCATTGAGCA	142
		GCTTGGTCACCACCATTGAG	
Hyaluronan and Proteoglycan Link protein 1 (HAPLN1)	AK313713.1	TGGTGAGAAAGTGCCTCCTT	151
		TAGCGCTCTTTCTCCTCACC	
Hyaluronan Synthase 3 (HAS3)	NM_005329.3	GGACCGTGTGCGGGATGTGG	142
		AGTGTCAGAGTCGCACACCTGGA	
Leptin (LEP)	NM_000230.3	GCTGTGCCCATCCAAAAAGTC	178
		CCAGTGTCTGGTCCATCTTGG	
Osteoprotegerin (OPG)	NM_002546.3	TGGCACCAAAGTAAACGCAGAG	195
		CTCGAAGGTGAGGTTAGCATGTC	
Osteopontin (OPN)	NM_000582.3	ACACATATGATGGCCGAGGTGA	115
		TGTGAGGTGATGTCCTCGTCTGT	
Periodontal Ligament-Associated Protein 1 (PLAP1)	NM_017680.5	ATGGGAGTCTTGCTAACATACCAC	154
		CAGAAGTCATTTACTCCCACTCTTG	
Periostin (POSTN)	NM_006475.2	CATTGATGGAGTGCCTGTGGA	167
		CAATGAATTTGGTGACCTTGGTG	
Runt-Related Transcription Factor 2 (RUNX2)	NM_001015051.4	AACCCTTAATTTGCACTGGGTCA	145
		CAAATTCCAGCAATGTTTGTGCTAC	
Semaphorin 3A (SEMA3A)	NM_006080.3	AACGGCCGTGGGAAGAGTCCAT	137
		TGGTGGTGCCCAAGAGTTCGG	
Tenomodulin (TNMD)	NM_022144.3	CTGGTGTTTGGTATCCTGGC	192
		ATCAGTGCCATTTCCGCTTC	

## Data Availability

Microarray data generated and/or analysed during the current study are available in the Gene Expression Omnibus (GEO) repository, Accession Number GSE235864. The other datasets for this study are available from the corresponding author upon reasonable request.

## Supplementary Materials

The following supporting information can be downloaded at: https://www.mdpi.com/article/10.3390/cells12232743/s1, Figure S1: Effects of various concentrations of HMWHA and LMWHA stimulation on COLA1 and POSTN expression in SK-PDLSCs. To determine the effects of hyaluronic acid molecular weights and its effective concentration on SK-N-SH cell differentiation, SK-N-SH cells cultured without HPDLSCs-ECM were treated with different concentrations (0.1, 0.3, 0.6, 1 mg/mL) of either high-molecular-weight hyaluronic acid (HMWHA) or with low-molecular-weight hyaluronic acid (LMWHA). After 2 weeks of continuous stimulation with HA, qPCR analysis of expression levels of *COL1A1* and *POSTN* was performed. A significant increase in the expression was noted in the samples treated with LHA with the concentration of 0.3 mg/mL showing the highest increase in *COL1A1* and *POSTN* expression. Meanwhile, the stimulation of SK-N-SH cells with HHA had almost no effect on the *COL1A1* and *POSTN* expression. Based on these results, we opted to use LMWHA at a 0.3 mg/mL concentration in further experiments.
