# Supplementary material for: Hyaluronic Acid Induction Promotes the Differentiation of Human Neural Crest-like Cells into Periodontal Ligament Stem-like Cells
Source: Cells. 2023 Nov 30;12(23):2743. doi: 10.3390/cells12232743 (PMC10705959; doi:10.3390/cells12232743)
Supplement: Supplementary file 1 [file cells-12-02743-s001.zip › cells-2688497-supplementary.pdf]

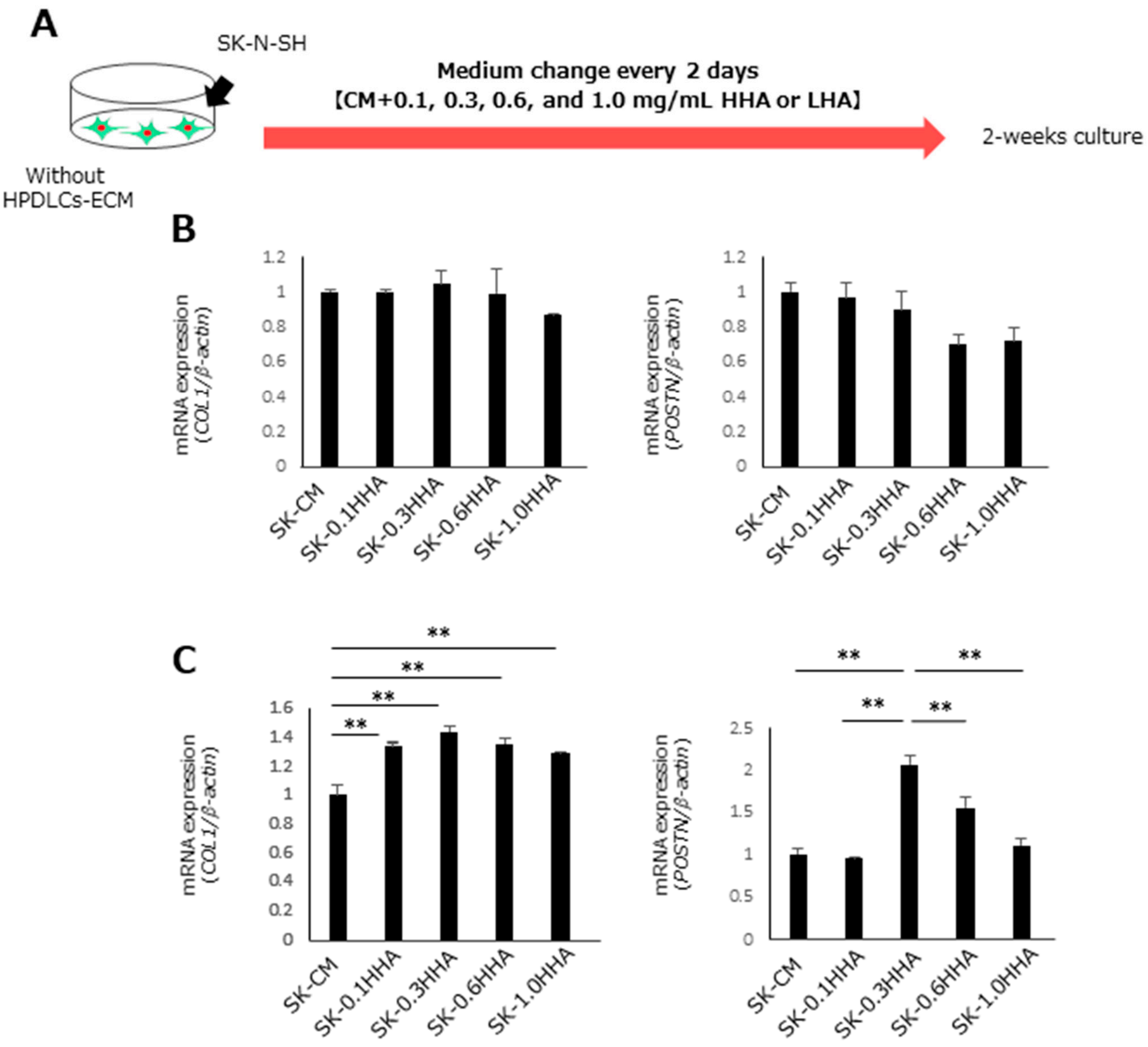

Supplemental Figure S1. Effects of various concentrations of HMWHA and LMWHA stimulation on *COLA1* and *POSTN* expression in SKN-SH cells.

(A) Schematic diagram of continuous stimulation with various concentrations of HMWHA and LMWHA in SK-N-SH cells cultured without HPDLSCs-ECM. (B, C) qPCR analysis of expression levels of *COL1A1* and *POSTN* in SK-N-SH cells cultured in CM containing various concentrations of HMWHA (B) and LMWHA (C). *β-actin* was used as an internal standard. Data expressed as the mean ± standard deviation (n = 3); $^{**}p < 0.01$. SK-N-SH+0.1HMWHA, SK-N-SH cells cultured with continuous 0.1 mg/mL HMWHA stimulation; SK-N-SH+0.3HMWHA, SK-N-SH cells cultured with continuous 0.3 mg/mL HMWHA stimulation; SK-N-SH+0.6HMWHA, SK-N-SH cells cultured with continuous 0.6 mg/mL HMWHA stimulation; SK-N-SH+1.0HMWHA, SK-N-SH cells cultured with continuous 1.0 mg/mL HMWHA stimulation; SK-N-SH+0.1LMWHA, SK-N-SH cells cultured with continuous 0.1 mg/mL LMWHA stimulation; SK-N-SH+0.3LMWHA, SK-N-SH cells cultured with continuous 0.3 mg/mL LMWHA stimulation; SK-N-SH+0.6LMWHA, SK-N-SH cells cultured with continuous 0.6 mg/mL LMWHA stimulation; SK-N-SH+1.0LMWHA, SK-N-SH cells cultured with continuous 1.0 mg/mL LMWHA stimulation.
